# Sociotechnical imaginaries of low-carbon waste-energy futures: UK techno-market fixes displacing public accountability

**DOI:** 10.1177/0306312720905084

**Published:** 2020-02-20

**Authors:** Les Levidow, Sujatha Raman

**Affiliations:** Open University, UK; Australian National University, Australia

**Keywords:** anaerobic digestion, bioenergy, mechanical and biological treatment, sociotechnical imaginaries, techno-fixes, UK low-carbon strategy

## Abstract

To implement EU climate policy, the UK’s New Labour government (1997–2010) elaborated an ecomodernist policy framework. It promoted technological innovation to provide low-carbon renewable energy, especially by treating waste as a resource. This framework discursively accommodated rival sociotechnical imaginaries, understood as visions of feasible and desirable futures available through technoscientific development. According to the dominant imaginary, techno-market fixes stimulate low-carbon technologies by making current centralized systems more resource-efficient (as promoted by industry incumbents). According to the alternative eco-localization imaginary, a shift to low-carbon systems should instead localize resource flows, output uses and institutional responsibility (as promoted by civil society groups). The UK government policy framework gained political authority by accommodating both imaginaries. As we show by drawing on three case studies, the realization of both imaginaries depended on institutional changes and material-economic resources of distinctive kinds. In practice, financial incentives drove technological design towards trajectories that favour the dominant sociotechnical imaginary, while marginalizing the eco-localization imaginary and its environmental benefits. The ecomodernist policy framework relegates responsibility to anonymous markets, thus displacing public accountability of the state and industry. These dynamics indicate the need for STS research on how alternative sociotechnical imaginaries mobilize support for their realization, rather than be absorbed into the dominant imaginary.

## Introduction: Techno-fixes as policy agendas

Debates about climate change pit incumbent high-carbon systems against more desirable low-carbon replacements. Amidst these contending agendas for technological change, some proposals – such as geoengineering, biofuels, and carbon capture and storage (CCS) – have been criticized by civil society groups as ‘techno-fixes’, even as ‘false solutions’ that perpetuate ‘carbon lock-in’ (Corporate Watch, 2008, 2019; ETC Group, 2015; FoEE, 2015). Such criticisms have been theorized by academics from various perspectives (e.g. Luke, 2010; Markusson et al., 2017; Unruh and Carillo-Hermosilla, 2006). Despite such criticisms, techno-fix agendas remain prevalent in efforts to address global challenges such as climate change.

The techno-fix concept originated from recognizing the dilemma of complex societal problems. Alvin Weinberg, former director of Oak Ridge National Laboratory, coined the term ‘technological fix’ as a more feasible alternative to ‘social engineering’ (Johnston, 2018). He asked: ‘Can we identify quick technological fixes for profound and almost infinitely complicated social problems?’ (Weinberg, 1966: 4; also 1995).

Techno-fixes have been long criticized as over-simplistic solutions to complex problems (Rosner, 2004). In similar terms, they are seen as hubris in the face of ‘wicked problems’ whose contradictory or unstable requirements cannot have a single solution (Hulme, 2014). The technofix pattern has been critically analyzed as ‘technological solutionism’, which presumes rather than investigates the specific problem it is trying to solve. In an idealized version, the correct combination of computer codes, algorithms and robots can solve all our problems (Morozov, 2013). Nevertheless, techno-fixes are attractive solutions for decision-makers seeking avoid responsibility for various industrial or political problems.

Many critiques have counterposed the need for social and institutional change beyond or alongside technological change, on several grounds. Science-led technological innovation has a limited capacity to fix societal problems, which instead warrant ‘social policy’ (Sarewitz, 1996). Problem complexity is why techno-fixes generally don’t succeed, except in the most straightforward cases (Nelson and Sarewitz, 2008).

Despite their divergence on the limitations of techno-fixes, both advocates and critics imply a clear distinction between social and technological change. This can be simplistic if presuming a choice between a social or technological intervention. The latter is always sociotechnical, involving societal order, often change.

Techno-fix agendas likewise have an ambiguous relationship to policy change. They have helped justify some shifts in policy, while serving to avoid others (as with biofuels, Levidow and Papaioannou, 2014, 2016; Raman et al., 2015). The latter role also can be seen in policy agendas for low-carbon technologies. At a global and national level, states have promoted market instruments as a necessary means to stimulate such technologies.

This market-driven agenda has been personified by Nicholas Stern since his report, *The Economics of Climate Change*, emphasizing that ‘policy must promote sound market signals’ for low-carbon development (Stern, 2006: i). He has advocated ‘radical change in approaches to cities, energy systems and land use’, especially through technological change (Stern, 2015: 6). From his diagnosis of market failure, Stern has advocated carbon markets as an arbiter of low-carbon technologies: ‘We should have a very open-minded attitude to technology and *let the markets decide* which to choose, without putting obstacles in the way that might arise from an antipathy to a particular technology’ (Stern, 2008, our emphasis).

According to critics, this policy framework displaces responsibility: ‘Many governments are meanwhile hoping that major climate investment decisions can be simply left to the new carbon markets’ (Lohmann, 2009: 1064). In emissions trading schemes, agents’ responsibilities are determined by daily market prices rather than by relevant governmental agencies (Page, 2012: 938, 940). Such policy frameworks pervasively rely on market instruments for technological solutions, while appealing to market competitiveness as means and ends. For this reason, we characterize the prevalent policy framework as techno-market fixes.

Anticipatory governance of new technologies could be extended to techno-market fixes as a means to gain public accountability. Anticipatory governance has been defined as 'a broad-based capacity extended through society that can act on a variety of inputs to manage emerging knowledge-based technologies while such management is still possible'. This approach has elicited some scepticism, for example, that technoscience may render anticipatory governance complicit in its hubris (Guston, 2014: 218). Indeed, state-led processes of anticipatory governance may end up conferring epistemic authority upon some technoscience-based visions rather than others.

To explore techno-market fixes, this paper compares how the UK government has promoted technoscientific solutions for bioenergy and for waste-energy issues. To analyse the latter in greater detail, this article discusses the following questions: What have been the different visions of societal futures? How did each one link technological solutions with institutional arrangements (change or continuity)? How did the UK policy framework relate to the different visions? How did anticipatory efforts gain epistemic authority for some visions rather than others? How did waste-energy outcomes relate to earlier promises of benefits, and with what accountability?

To answer these questions, this paper draws on the theoretical framework of sociotechnical imaginaries, as elaborated in the next section (Jasanoff, 2015; Jasanoff and Kim, 2009). The discussion of sociotechnical imaginaries will help illuminate how the UK’s techno-fixes rest on a particular ecomodernist policy framework, which we discuss in the subsequent section. This theoretical linkage enables us to compare rival imaginaries for UK low-carbon trajectories, as summarized in Table 1 and our conclusion. The comparison highlights continuities in policy narratives regarding societal visions, techno-market fixes and epistemic reasoning.

**Table 1.** Rival imaginaries of a low-carbon future from biomass or waste. The lower part corresponds to the waste-conversion case studies.

	Dominant: techno-market fix	Alternative: eco-localization
Stakeholder groups	Large energy and waste-management companies; technology suppliers; government Ministries	*Zero Carbon Britain* (CAT, 2007), Campaign Against Climate Change, Friends of the Earth (FoE), Transition Towns
Public good: substitutes for fossil fuels	Low-carbon technologies will more efficiently convert feedstock as inputs to centralized systems, thus greening them.	Low-carbon systems (energy, agriculture, transport) should minimize resource burdens, localize resource flows and diversify output uses.
Socio-political order	Let the market decide on optimal techno-trajectories in response to financial incentives and penalties.	Establish support measures for localizing institutional responsibility for low-carbon systems.
Policy incentives most favourable	PFI waste infrastructure subsidy drives investment in large energy-from-waste (EfW) plants. Renewable Obligation drives large-scale electricity generation.	PFI programme incentivizes large, inflexible facilities rather than maximize recycling. Feed-in-Tariff has incentivized small-scale plants, but the tariff declined after 2010. The Renewable Heat Incentive was meant to expand heat use – but inadequate to incentivize new infrastructure for heat distribution.
‘Sustainable’ biomass usage	Residual or sustainable biomass, defined in a broad way, is conversed to energy as input-substitutes for fossil fuels.	Biomass is valuable resource for recycling or carbon sequestration in the soil, only exceptionally for energy production (e.g. woody plants).
2^nd^ generation biofuels (non-edible feedstock)	R&D priorities envisage 2G fuels as large-scale input-substitutes for fossil fuels, dependent on mandatory quotas	Mandatory quotas may lock-in conventional biofuels and perpetuate the internal combustion engine.
**Waste feedstock for conversion**		
Energy-from-waste (EfW) plants for municipal solid waste	EfW outputs can go to gas or electricity grids anywhere as a global good.	EfW plants waste resources and make little use of their surplus heat.
Epistemic authority	Know-how for maximizing waste-based energy production to substitute for fossil fuels	Know-how for bringing waste up the hierarchy through conversion processes and output uses
AD roles (optimal)	Large-scale biogas production (energy company vision). Larger-scale plants diversify feedstock sources to maximize subsidy income	On-farm waste management (farmer and NGO vision) with biogas for local use.
AD feedstock sources (optimal)	Food waste can be supplemented by maize to stabilize electricity production	On-farm plants convert animal slurry, which otherwise would pose an environmental burden, while locally using all outputs. Maize feedstock worsens environmental burdens.
AD: uses of digestate and heat	Plant operators pay a gate fee to spread low-mineral digestate on farms. Concentrating the mineral content could create a widely transportable product.	On-farm digestate, familiar to each farmer, readily substitutes for chemical fertilizer. Find nearby uses also for surplus heat.
MBT design and trajectory	MBT to generate RDF for EfW plants → electricity substituting for fossil fuels → a global good (energy company vision)	MBT biostabilization plants will generate minimize methane emissions, significantly reduce output volume and produce a Compost-Like Output (CLO) as soil improver (NGO vision).
MBT operation in practice	RDF-to-EfW plants produce energy as global good, saving GHG emissions in relation to fossil fuels.	RDF-to-EfW plants generate more net GHG emissions than the landfill option. Yet MBT-CLO plants have had operational difficulties for reliable outputs.

## Methods

This paper draws on the three UK research projects. Each project reviewed relevant academic, policy, industry, civil society, and trade press literature. Documents were analysed for assumptions, visions or expectations about several aspects of the research topic, including: technological innovation, environmental sustainability, feedstock sustainability, feedstock conversion, market incentives, waste hierarchy, operational scale, and responsibilities. We then conducted interviews based on findings from the document review.

Our retrospective analysis draws on theoretical perspectives about how ‘technology in use’ undergoes adaptive adjustments (Edgerton, 2006). We compared earlier anticipations with outcomes a decade later, paying particular attention to changes in priorities and technoscientific designs. In doing so, we follow Hyysalo et al. (2018: 10) who note: ‘There is an opportunity to extend enquiry longitudinally – which may serve to increase our robustness of understanding of innovation processes and their outcomes.’ However, it was difficult to identify key individuals still involved from a decade earlier; hence, our historical data depend mainly on documentary sources.

## Sociotechnical imaginaries construing futures: Analytical perspectives

Imaginaries denote desired or construed futures. They can ‘guide a critical mass of self-confirming actions premised on their validity’, thus constituting a future world (Jessop, 2010: 338). Critical analysis seeks ‘to explain why and how some construals are selected, get embodied in individual agents or are routinized in organizational operations’ (Jessop, 2010: 339), institutionalizing specific practices in the process.

As understood by cultural political economy, an ‘imagined economic space’ may become grounded in an ‘imagined community of economic interest’ (Jessop, 2005: 162). In order to assemble effective coalitions, actors ‘articulate strategies, projects and visions oriented to these imagined economies’ (Jessop, 2010: 345). Economic imaginaries often frame territorial jurisdictions as competitive units in an economic fight with foreign rivals. For example, ‘Europe’ becomes a single political-economic competitive space facing a common external threat (Rosamond, 2002: 169). In their performative role, imaginaries serve to mobilize economic resources, thus creating the conditions to achieve specific futures. Similar concepts are developed in STS regarding how ‘sociotechnical imaginaries’ articulate ‘the relationship of science and technology to political institutions’ (Jasanoff and Kim, 2009: 120).

### Dominant sociotechnical imaginaries

An early definition of sociotechnical imaginaries describes them as ‘collectively imagined forms of social life and social order reflected in the design and fulfillment of nation-specific scientific and/or technological projects’ (Jasanoff and Kim, 2009: 120). Sociotechnical imaginaries describe what constitutes the public good or a good life, especially as promoted by a state agency. Such an imaginary is ‘an important cultural resource that enables new forms of life by projecting positive goals and seeking to attain them’ (Jasanoff and Kim, 2009: 122). In this way, they can inform and justify innovation policies:

Such policies balance distinctive national visions of desirable futures driven by science and technology against fears of either not realizing those futures or causing unintended harm in the pursuit of technological advances. S&T policies thus provide unique sites for exploring the role of political culture and practices in stabilizing particular imaginaries. (Jasanoff and Kim, 2009: 121)

An imaginary may serve to mobilize various organizations and resources, shaping ‘the hearts and minds of human agents and institutions’ level (Jasanoff, 2015: 17).

By turning to sociotechnical imaginaries, we can engage directly with the ways in which people’s hopes and desires for the future — their sense of self and their passion for how things ought to be — get bound up with the hard stuff of past achievements, whether the material infrastructures of roads, power plants, and the security state, or the normative infrastructures of constitutional principles, juridical practices, and public reason. (Jasanoff, 2015: 22)

The concept helps to understand ‘why different moral valences attach to new scientific ideas and technological inventions’; likewise ‘how actors with authority to shape the public imagination construct stories of progress in their programmatic statements, and how they blend into these their expectations of science and technology’ (Jasanoff, 2015: 337). Conversely, imaginaries can help states to gain authority for exercising power (Jasanoff and Kim, 2009: 123). The concept helps to explain ‘how technological and political orders are co-produced’ in distinctive ways (Jasanoff and Kim, 2009: 124). Any national debate reinforces ‘patterns of public reason, evidence production and knowledge uptake that constitute a nation’s political culture’ (Jasanoff and Kim, 2009: 140).

Although early formulations emphasize nation-states as key actors, others can originate visions that become communally adopted: ‘Multiple imaginaries can coexist within a society in tension or in a productive dialectical relationship’ (Jasanoff and Kim, 2015: 4). This signals key questions: How do multiple sociotechnical imaginaries envisage the public good in conflicting ways, and how do these play out in effect? A critical approach needs to analyse state-level narratives of the ‘good life’, especially whether they are ‘collectively imagined’ by the entire society or else face rival visions of the future (Tidwell and Tidwell, 2018: 104). This perspective pushes researchers to analyse how an imaginary becomes contentious, as elaborated next.

### Ecomodernist context of rival imaginaries

In ecomodernist policy frameworks, states aim to stimulate the self-regulation of industry, thus transferring responsibilities from the state to the market (Mol, 1996: 306). Through market-based instruments such as environmental taxes, the ‘greening’ of industry involves a process of ‘economizing ecology’, or attributing economic value to environmental resources and burdens (Mol, 1997: 141). Such agendas promote techno-fixes and justify institutional changes to facilitate them:

[Ecomodernism] uses the language of business and conceptualizes environmental pollution as a matter of inefficiency, while operating within the boundaries of cost-effectiveness and administrative efficiency … [Ecomodernism] is … basically a modernist and technocratic approach to the environment that suggests that there is a techno-institutionalist fix for the present problems (Hajer, 1995: 31–32).

Since the 1990s, policy frameworks have promoted techno-fix agendas for market-driven eco-efficiency gains. The analytical concept ‘techno-market imaginary’ describes such frameworks that promote innovation, entrepreneurship, venture capital and carbon markets. The techno-market imaginary ‘allocates a primary role to the private sector in addressing climate change, lending this imaginary a broad appeal across multiple constituencies’, which include financial interests competing on global carbon markets (Levy and Spicer, 2013: 664, 669). This concept links the ecomodernist perspective with cultural political economy.

The techno-market imaginary … assumes that the environment is somewhat vulnerable, but that the climate issue is manageable through appropriate economic incentives and technological innovation, without fundamentally compromising lifestyles or economic growth. This imaginary’s positioning highlights its hegemonic appeal, by claiming to reconcile economic and environmental concerns (Levy and Spicer, 2013: 666).

Hence, the techno-market imaginary has gained policy dominance over rival imaginaries, for example, ‘fossil fuels forever’ and ‘sustainable lifestyles’, though they still maintain some support.

As a contentious case in many countries, state authorities have incentivized the expansion of biofuels with feedstock from edible biomass and/or waste wood. In Michigan, for example, a sociotechnical imaginary anticipated efficient bioenergy plants providing cleaner, renewable and therefore sustainable energy production. This framed woody biomass as residual, dispensable and thus sustainably available. By contrast, some local views were more cautious about its environmental sustainability. Nevertheless the state imaginary expressed ‘confidence, authority, credibility, and a capacity to tame and control any wickedness bioenergy may pose’ (Eaton et al., 2014: 250).

In EU and some national imaginaries of biofuels, market incentives stimulate technoscientific advances for eco-efficiency improvements and competitive exports. According to the UK’s Renewable Energy Strategy, UK biofuel producers ‘will have the opportunity to compete in a global market if they can meet the European mandatory standards’ (DECC, 2009a: 111). This imaginary provided a response to controversy over the environmental and social sustainability of early biofuels. They were retrospectively renamed ‘conventional’ or ‘first-generation’. This implied a temporary role in a transition to second-generation and hence more advanced biofuels using waste or non-food feedstock, thereby avoiding harm from conventional biofuels.

Invoking such a future transition, the European Union (among other state authorities) mandated statutory quotas for renewable energy in all transport fuels (EC, 2009). The only short-term option was conventional biofuels, so the quota stimulated a market that otherwise would not exist. It gained some epistemic authority from the promised advances; yet these have remained elusive, thus perpetuating the current fuel system:

This powerful strategy aims to project the illusion of radical change while concealing that the envisioned technological developments strive to maintain the status quo rather than altering it to achieve a better and greener future. In this sense the sociotechnical imaginaries of advanced biofuels, provided through the epistemic authority of international organizations, are devoid of utopian potential (Kuchler, 2014: 436).

Like the EU, the UK’s sociotechnical imaginary anticipated that second-generation biofuels would eventually link environmental sustainability with competitive advantage, as a basis for greening transport fuel. These epistemic claims depended on specific models of GHG savings from various non-edible feedstock for second-generation biofuel plants. Such accounting framed all such resources as ‘waste’, that is as a burden lacking other potential uses; this normative assumption underlay calculations justifying waste conversion to energy for lower GHG emissions. On this basis, the dominant imaginary promoted ‘institutional change that reinforces infrastructural dependence on liquid fuel for the internal combustion engine’ (Levidow and Papaioannou, 2014: 280). For these reasons, the UK’s statutory quota for biofuels became increasingly controversial.

Beyond Western countries, we can see analogous cases of techno-market fixes. In Thailand, for example, rival sociotechnical imaginaries that intertwine with political economy in different ways are a defining characteristic of recent energy transitions. The dominant imaginary advocates the energy security of Thai industry, and thus increasing Thailand’s economic competitiveness, through continued economic growth, state financial incentives, and the combined use of renewables and coal. At the same time, however, an alternative imaginary rooted in civil society questions market expansion, asking about who will benefit from increased energy. It prioritizes ‘sustainable development as a social and environmental project of sustaining lives without hampering the ability of nature to replenish itself’ (Delina, 2018: 54). This alternative advocates the decoupling of economic growth from fossil fuel consumption, greater equity in the use of resources, better livelihoods, and decentralized energy solutions, for example, by helping ordinary Thai households to generate electricity through rooftop solar panels (Delina, 2018). The latter vision can be seen as an eco-localization imaginary.

After Japan’s 2011 nuclear disaster, Fukushima Prefectura proposed alternatives to the country’s national energy plan, thus generating rival imaginaries. They differ on many issues, especially on how hydrogen should be deployed as either part of Japan’s centralized energy system or part of its transition to a locally distributed renewables-based society. The former agenda invokes global market imperatives. ‘Economically oriented rhetoric in the national hydrogen imaginary portrays ambitions of maintaining Japan’s global leadership in hydrogen and fuel-cell technologies in the interests of maintaining international competitivity in a niche but rapidly growing global market’ (Trencher and van der Heijden, 2019: 217). Japan’s rival imaginaries have been somewhat accommodated by the flexible design of hydrogen technology:

since hydrogen can be produced from both fossil fuels and renewables through either centralised or decentralised energy systems, the imaginary of a hydrogen future is able to generate support from fossil fuel incumbents in favour of a centralised energy system … just as much as from renewable energy protagonists in favour of decentralised models of local production and consumption. (Trencher and van der Heijden, 2019: 210)

Hydrogen futures have an interpretive flexibility that broadens their appeal to actors with diverse expectations, values and expertise (cf. Eames et al., 2006).

These examples illustrate how environmental problems are framed in divergent ways through rival imaginaries, especially techno-market versus eco-localization ones (see also Feola and Jaworska, 2018; Levidow and Papaioannou, 2016; North, 2010). Ecomodernist policy frameworks can discursively accommodate these imaginaries, perhaps softening tensions between them. As the dominant imaginary, techno-market fixes appeal to an economic imaginary (Jessop, 2005), especially the nation as a single competitive space facing a common external threat and market opportunity (Rosamond, 2002).

In the name of innovative solutions, moreover, techno-fixes can help avoid systemic change. This role has a long history:

Calling for innovation is, paradoxically, a common way of avoiding change when change is not wanted. The argument that future science and technology will deal with global warming is an instance. It is implicitly arguing that, in today’s world, only what we have is possible. (Edgerton, 2006: 210)

More broadly, regardless of whether a significant change is wanted by some publics, a techno-fix can reinforce dominant production-consumption systems.

## The UK ecomodernist framework for a low-carbon strategy

Within the EU ecomodernist framework, the UK’s dominant sociotechnical imaginary envisions centralized systems as the most efficient and cost-effective strategy for transitioning to a low-carbon future. At the same time, the UK’s low-carbon strategy features different visions about technoscientific innovation helping to localize systems for greater environmental benefits. These dual imaginaries co-exist in tension, as we show below in the example of renewable energy and a more detailed analysis of waste-conversion trajectories. (See the upper half of Table 1.)

### Renewable energy fixes

For its low-carbon strategy, the New Labour government (1997–2010) stimulated new markets for emerging technologies through several policies including research and development funds, landfill tax and market quotas. Under the Renewables Obligation, electricity suppliers had to source ten percent of their electricity from renewable sources by 2010. By relying on market mechanisms, the government tried to ‘bolt environmental goals onto its existing economic strategies’ (Revell, 2005: 358). But market-type instruments could not resolve the tensions between economic and environmental objectives.

Alongside the dominant imaginary of centralized systems, government policy included an imaginary of both localizing and decarbonizing energy supplies for multiple public benefits. For example, the 2003 Energy White Paper emphasized greater use of biomass for combined heat and power, especially for small-scale local uses. The document promoted multiple bioenergy sources as important components ‘in widening fuel diversity and energy security in the transport sector’ (DTI, 2003: 69).

Later UK reports reiterated an eco-localization imaginary: ‘a combination of new and existing technologies are opening up new possibilities for carbon reduction by producing and using heat and electricity at a local level, that is, distributed or decentralised energy’ (DTI, 2007: 12). ‘A further factor that is likely to increase the economic favourability of bioenergy is the decentralisation of power generation through microgeneration’, according to the UK Energy Research Centre (UKERC, 2009: 3). Energy localization depends on local communities. Therefore, for renewable energy in general, UK strategy should attempt ‘to ensure stronger local participation in projects, and sharing of benefits via local communities’, according to the statutory Committee on Climate Change (CCC, 2011: 106).

In parallel, the government went beyond EU targets for greenhouse gas reductions. The 2008 UK Climate Change Act set a 2050 target date for a statutory duty to reduce greenhouse gas emissions by 80%, relative to the 1990 baseline. To fulfil this target, the government sought ‘a secure, low-carbon future’. Its strategy sought to decarbonize the electricity sector, as a step towards substituting electricity for fossil fuels. Most remedies invoke the key term ‘efficiency’, that is technologies more efficiently producing or using energy, especially from renewable sources. In particular, ‘[w]e will also need a bigger, smarter electricity grid’, argued the Department of Energy and Climate Change (DECC, 2009b: 10); this centralized system was seen as relatively more efficient.

The government’s policy framework for a low-carbon transition sought to reconcile environmental aims with dominant economic interests. It positioned itself as proactive with regard to climate change, constantly engaging and building ‘partnerships’ with industrial and other non-governmental actors, thus blurring responsibility for solutions (Carvalho, 2005: 15). Various pro-industry policies were addressing climate change, while also pursuing economic aims: ‘climate change was subsumed in wider agendas and was often used to justify externally-motivated measures’ (Carvalho, 2005: 19–20). Climate protection has been the putative rationale for policies that prioritize other aims, especially economic growth via low-carbon industry. The ‘further rapid growth’ of the sector has been advocated by the Department of Energy and Climate Change (DECC, 2009b: 61).

Tensions arose between sociotechnical imaginaries for bioenergy in particular. Civil society alliances had long advocated a transition that would decentralize renewable energy and reduce energy demand across several sectors. In the eco-localization imaginary, biomass had the modest roles of recycling natural resources and sequestering carbon. By contrast, the dominant imaginary promoted biomass conversion as an input-substitute for fossil fuels within centralized energy systems; technoscientific innovation would convert lignocellulose through substantial inputs of energy and water (Levidow and Papaioannou, 2016).

The eco-localization imaginary was elaborated in the *Zero Carbon Britain 2030* report. It advocated government policies that would help create a market for low-energy, low-carbon technologies (CAT, 2007). Dominant assumptions about energy efficiency were inverted: small-scale renewables ‘help increase efficiency and decrease demand’ (CAT, 2010: 16). Its agenda gained support from the Campaign Against Climate Change but no national political mobilization to obtain the necessary policy instruments and economic resources.

Some community initiatives have set up locally owned production of renewable energy, especially in areas distant from the national grid. Some urban initiatives also have pursued such a transition pathway, but they tend to encounter greater obstacles:

These [UK alternative] pathways focus less on ‘upstream’ large-scale technologies and more on reconfiguring local energy and transport systems. These alternative transition pathways receive less attention and resources, which shows that the dominant prognostic discourse privileges the interests of centralized incumbent actors rather than those of less organized and local actors. (Geels, 2014: 32)

The government promoted financial incentives meant to stimulate eco-efficient technoscientific innovations. Such programmes were intended to foster lower-carbon trajectories, substitute renewable energy for fossil fuels, and reduce waste or use it in environmentally beneficial ways. Policy incentives have been conceptually justified by ‘market failure’, to be corrected by support measures for ensuring fair market competition and thus technological innovation:

Innovative renewable technologies face many barriers to their development and successful commercialisation, and the Government has a fundamental role in setting frameworks in which markets can operate fairly and effectively to help the private sector bring technologies through to large-scale deployment. (HM Govt, 2009: 136)

Indeed, the UK government has claimed to be technology-neutral by ‘allowing the market to decide’ innovation priorities amongst low-carbon options (cited in Levidow and Papaioannou, 2016). Within this policy framework, short-term policy commitments have presumed that specific technologies would become competitively self-financing. From 2010 onwards, feed-in-tariffs targeted small-scale electricity generation at levels <5 MW from solar, offshore wind and bioenergy. This financial incentive was advocated by environmental NGOs as means to expand renewable energy sources, especially in decentralized forms (Toke, 2012).

From the government perspective, however, the tariffs aimed to establish the commercial viability of new technologies. On such grounds, after 2010 the tariff levels underwent a stepwise decline. This made new small-scale investments too risky, while leaving incumbent energy companies to incorporate such technologies into their large-scale systems.

As a different support measure, from 2002 onwards the Renewables Obligation required energy suppliers to supply increasing proportions of electricity from renewable sources and to gain validation through Renewable Obligation Certificates. But the Renewables Obligation focused on large-scale generation at capacity above 5 MW. Also, the focus on electricity ignored heat-only applications. This incentivized an expansion of bioenergy plants, initially with edible biomass and/or woodchips, sometimes co-fired with coal.

Large-scale biomass conversion became an imperative in the UK’s dominant sociotechnical imaginary. This framed ‘sustainable biomass’ in a broad way (e.g. woodchips from North America) as a renewable input-substitute for centralized systems. Efficient conversion was nearly equated with environmental sustainability: 2G biofuels ‘should make the production of biofuels from land much more efficient, with a reduced area needed to produce a given volume of biofuels’, declared the Department of the Environment and Rural Affairs (DEFRA, 2007a: 22, 36).

The policy narrative warned against locking-in environmentally sub-optimal pathways, yet these were framed narrowly as problematic feedstock sources (e.g. edible biomass or biomass-coal co-firing). They became a temporary, transitional step towards more a sustainable future and thus not a lock-in. Moreover, the public good was equated with current infrastructure. According to industry stakeholders, biofuels as input-substitutes help to protect the investment value of the current transport-energy infrastructure, as well as consumer freedom through private motor vehicles. Thus the dominant imaginary reinforced incumbent energy companies and infrastructural dependence on liquid fuel for the internal combustion engine (Levidow and Papaioannou, 2014).

Analogous tensions between dual imaginaries arose in techno-trajectories for treating waste as a resource, as shown in subsequent sections.

### Waste-conversion fixes

Throughout the EU, waste-management systems have been undergoing pressures to move beyond mere disposal, especially landfill, whose methane emissions are a potent greenhouse gas. As well as reducing greenhouse gas emissions and waste generation, EU policy has promoted various means to recover resources to substitute for products dependent on fossil fuels. The framework outsources the task to the private sector, which is then expected to deal with such tensions:

Municipal waste-management companies perform the fix. They operate in institutional environments that sometimes allow them to act on markets but in other instances prevent them using the full potential of their material management competence and infrastructure in an economically competitive manner. (Hultman and Corvellec, 2012: 2418)

**Figure 1.**
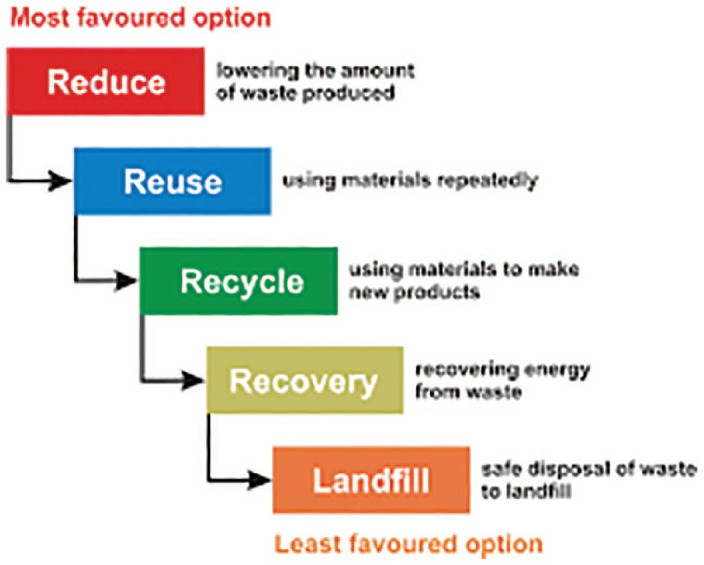
Waste hierarchy. credit: DEFRA.

The EU formalized the waste hierarchy with its priorities: prevent, reduce, reuse, recycle or recover waste. The 2008 EC Waste Framework Directive ‘brings a modernised approach to waste management, marking a shift away from thinking about waste as an unwanted burden to seeing it as a valued resource’ (CEC, 2010: 5; also DEFRA, 2011a; EC, 2008). The waste hierarchy integrates the ‘alternatives of reducing waste and extracting value from it’ (Corvellec and Hultman, 2011: 5–6) (Figure 1).

Decisions on waste-treatment plants have faced several intersecting pressures. In the EU policy framework, several measures sought to move waste away from disposal. The EC Landfill Directive (1999/31/EC) obliges Member States to reduce the amount of biodegradable municipal waste going to landfill to 50% of 1995 levels by 2013 and 35% by 2016 – or by 2020 for some countries, including the UK (EC, 1999).

Within the EU framework, UK policy has long promoted the waste hierarchy as a ‘guiding principle’ for new facilities (DEFRA, 2007b: 2). The policy links environment with economy: ‘The dividends of applying the waste hierarchy will not just be environmental. We can save money by making products with fewer natural resources, and we can reduce the costs of waste treatment and disposal’ (DEFRA, 2007c: 9).

Alongside renewable energy, the UK government has sought to reduce greenhouse gas emissions from waste-management practices through joint solutions with industry. Implementing the EC Landfill Directive, the UK’s Landfill Tax Escalator set a timetable for annual tax increases that rose sharply starting in 2005 and quadrupling in the subsequent decade; this drove up the gate fees paid to waste-management companies. This rise stimulated efforts towards waste reduction and kerbside segregation, which in turn facilitated recycling and organics segregation; the latter provides inputs for a composting process or anaerobic digestion plants.

Despite efforts at segregating components of municipal solid waste, large quantities still need an outlet. UK policy promoted waste-to-energy plants: ‘Generating renewable energy from biomass waste could also significantly reduce the amount of waste that is landfilled in the UK’ (HM Govt, 2009: 87, 104). When more waste was diverted to new incinerators, however, they often faced public opposition. Some protests demanded alternatives to minimize waste transport (Dodds and Hopwood, 2006; Rootes, 2009). Although these protests rarely closed down plants, the conflict deterred many local authorities from commissioning new incinerators and pushed them towards alternatives with greater local responsibility, as shown in the two case studies that follow.

To go beyond conventional energy-from-waste (EfW) plants, UK support measures stimulated private-sector investment in Advanced Thermal Treatments. In particular, a gasification process aimed to produce a clean syngas (synthetic gas) that could substitute for fossil fuels. Yet such experimental plants had great difficulties treating variable heterogeneous feedstock (Levidow and Upham, 2017). Regardless of the technology, civil society groups denounced all thermal processes for wasting resources and undermining pressures to minimize waste production (UKWIN, 2010). These critics counterposed a circular economy to restructure and localize production processes, reducing waste (UKWIN, 2016).

Those rival visions were subjected to the market-efficiency rationale of the UK framework: ‘Government policy is driven by the desire to drive waste up the hierarchy’ (DEFRA, 2014: 67). As the underlying diagnosis of the problem, market failures generate environmental externalities, warranting policy intervention. For the waste-management sector, for example: ‘Ensuring that the amount of waste is reduced to the economically efficient level, and is optimally managed, will ensure that waste policy is delivering net benefits for society as a whole’ (DEFRA, 2011b: 4, 8). This policy framework has sought temporary adjustments in markets so that they keep waste within economically-environmentally optimal levels and facilitate its conversion.

Market adjustments have meant to incentivize industry’s task, namely: matching technology appropriately with feedstock in order to move waste up the hierarchy. ‘There are a range of technologies for recovering energy from waste. It’s a question of matching the right technology with the right fuel, depending on the nature of the fuel and the desired outputs’ (WRAP, quoted in REA, 2011: 2). Yet in practice such ‘matching’ is often elusive.

UK policy has sought to stimulate new markets for technological improvements by treating waste as a resource, especially through renewable energy production. Creating the ‘right’ price signals for business became the favoured government means to address environmental challenges. Financial devices shaped the ability to re-scale waste by spatially distancing waste from its point of production and transforming it into a resource for energy (Reno, 2011).

UK policies have relied on financial incentives stimulating local authorities to privatize waste management. Consequently, new commercial markets turned waste into a resource for large EfW plants. Waste-treatment systems shifted to a larger scale that is distant from any specific end-use. Consequently, some claims for global goods turned out to be environmentally contentious or rivalrous, that is mutually incompatible demands on the same resource (Alexander and Reno, 2014: 351–4).

The above discussion illustrates tensions between techno-market and eco-localization imaginaries. The latter underlay environmental NGOs’ support for two waste-treatment technologies in particular: Anaerobic Digestion (AD) that processes organic waste into producing low-carbon biogas plus digestate as a potential fertilizer, and Mechanical and Biological Treatment (MBT) that processes municipal solid waste in various ways.

The two UK cases have analogous features. In both cases, the initial technology designs were meant to move waste up the hierarchy, gaining broad support on that basis. For each technology, two sub-sections below analyse rival imaginaries; together these correspond to the lower half of Table 1.

## Anaerobic Digestion: Dual sociotechnical imaginaries

**Figure 2.**
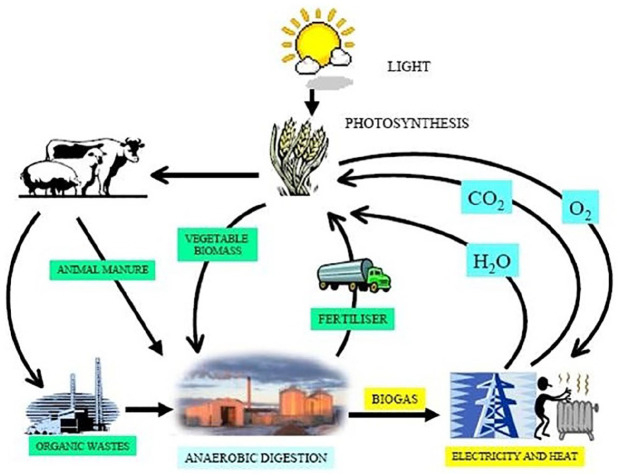
Anaerobic digestion, feedstock sources and carbon cycle (Wikid Energy Funhouse, 2013).

Anaerobic Digestion plants can process organic material of various kinds (Figure 2). An oxygen-free chamber induces the organic matter to break down, producing a methane-rich gas (biogas) and nutrient material (digestate). Biogas can be used as a source of heat for cooking, other types of food processing or space heating.

More complex, mature technological operations process biogas to produce heat and electricity in co-generation plants (combined-heat-and-power). Alternatively, they may purify and upgrade the biogas to natural gas standards (biomethane) for use in transport as a renewable alternative to fossil fuels. When outputs are connected to the electricity or gas grid, they must fulfil specific standards.

After the turn of the century, small-scale AD plants were being multiplied within an agenda to reduce and source-segregate organic waste. This can be understood as an eco-localization imaginary. However, financial instruments later shaped technological design towards supplying centralized energy systems; this has stimulated larger-scale, longer-distance material flows. These dual imaginaries are analysed in the next two sub-sections, respectively.

### Expanding AD for localized waste management

In the 1970s, the government began to see livestock waste as a pollution problem and legislated for its abatement. In the 1980s, it envisaged AD as enhancing the farm’s environmental sustainability (Ward et al., 1995). The government’s farm waste-management plans helped stimulate around 30 farm-based AD units from the late 1980s to 1995 (Bywater, 2011; Sanders et al., 2010). These were typically aided by grants for up to approximately half of the cost.

Digestate leftover from the AD process replaced the untreated slurry that was normally spread on soil, thus reducing the risk of pollution. AD also provided heat, which could be used on farms to run a boiler for heating farm buildings or providing hot water. In this context, waste was managed on-farm with the help of AD technology, adding benefits for farm sustainability.

The New Labour government’s eco-modernist approach reconceptualized waste as a resource for a low-carbon strategy. Its Biomass Strategy promoted AD for its wider potential to manage diverse wastes, as well to reduce greenhouse gas emissions, including those from manure, slurries and other biowaste. The government set out a vision to widely establish AD around the country by 2020, partly moving waste up the hierarchy (DEFRA et al., 2007). AD was conceptualized as a multi-functional technology which would help manage waste, generate energy, provide bio-fertilizer and offer the UK a competitive advantage in technology export. As a flexible multi-scalar technology, AD would be promoted at different scales (DEFRA, 2009).

Financial instruments were adjusted ‘to encourage a variety of energy recovery technologies (including anaerobic digestion) so that unavoidable residual waste is treated in the way which provides the greatest benefits to energy policy’ (DEFRA, 2007a: 14). One pre-existing mechanism was the landfill tax: ‘Increasing the tax to a higher level makes investments in alternative non-landfill treatments such as recycling and anaerobic digestion more economically viable’ (DEFRA, 2007a: 34).

By the end of the decade, financial incentives specifically aimed to stimulate ‘small-scale’ renewables through subsidy payments calculated by energy output, with smaller units receiving higher tariffs. First, the Renewable Heat Incentive rewarded renewable heat generators installed from 2009 onwards, providing a tariff payment (DECC, 2011). Second, after 2010, feed-in-tariffs targeted small-scale electricity generation at levels less than 5 MW.

Many stakeholder groups promoted future visions of localizing waste management and use, especially through AD (Levidow and Papaioannou, 2013, 2016). The AD programme had support from environmental NGOs, for example: ‘To minimise the impact our waste has on the climate, Friends of the Earth believes that compostable and recyclable material should be separated at source for treatment or reprocessing, using AD where suitable’ (FoE, 2007: 1).

Options for managing farm waste within farms or their neighbouring communities were essential to this future, as promoted by the Royal Agricultural Society of England (RASE, 2011). The National Farmers Union set out a vision of 1000 AD plants in the UK by 2020 with four different scalar models. They argued that regulatory criteria should not disadvantage single-farm or multi-farm community plants built with on-farm inputs and for on-farm use (NFU, 2009).

The feed-in-tariff scheme was meant to support small-scale installations, that is generating ‘less than 500 kW’. It incentivized many farm-based digesters at different sizes below 250 kW. Yet this ‘small’ classification neglected the challenges of generating even 500 kW with farm slurry and manure as the main feedstock. The average UK dairy farm has 100–130 cows producing slurry over 6–7 months, adequate for generating the biogas equivalent of only about 5 kWe (thermal equivalent for heat; Bywater, 2011). Converting this heat to electricity, rather than simply using the heat on-farm, requires extra equipment and costs, which in turn means reconfiguring AD along different scalar lines.

The government’s 2011 Action Plan for AD endorsed localizing waste management as well as energy generation. The Plan highlighted ‘significant potential for increasing uptake in England’ if barriers could be overcome (DEFRA and DECC, 2011: 2). Yet financial instruments alone could not overcome the many obstacles.

### Expanding AD for electricity supply

Going beyond the farm, the UK strategy sought to recover and use waste from diverse sources through large AD plants, stimulated by electricity subsidy. This amounts to a techno-market fix for production-consumption systems that generate waste. The government envisaged:

Putting even less of the waste we produce into landfills: The Government will encourage greater production of bioenergy, particularly from combustion. It also plans to encourage more processing of food waste, agricultural waste, and sewage using ‘anaerobic digestion’ to produce biogas. (DECC, 2009b: 5)

After 2002, the Renewables Obligation stimulated anaerobic digestion at the upper end of the plant-size spectrum, that is 250–500 kWe and even larger. AD plants have been upscaled in ways maximizing grid-fed electricity production. Through such market-based initiatives, ‘renewability focused on the farm is losing favour to new energy sources for the national grid’ (Reno, 2011). By building more AD plants, potentially beyond the capacity for feedstock collection (Eunomia, 2014), the system may lock-in a longer-term dependence on specific waste streams: ‘If AD plants are built, they need food waste collections in place. For these food waste collections to work, they need the public to continue wasting food – the very problem many are trying to stop’ (Clay, 2016: 14).

Upscaling AD plants also increases the use of non-waste feedstocks. Larger plants depend on collecting, transporting and converting more diverse sources of organic matter. To ensure a steady operation with high-energy output, some have become dependent on maize. Its cultivation had previously trebled between 1990 and 2000 for animal feed. A decade later its cultivation increased further in response to financial incentives for AD feedstock. The industry justified the rising usage for AD plants on several grounds, especially that maize cultivation does not displace food crops and is more energy-efficient than other bioenergy crops, according to the Anaerobic Digestion and Biogas Association (ADBA, 2011: 5–6; More, 2017; see again Figure 2). Maize for AD feedstock sharply increased after 2012, soon reaching one-fifth of all maize cultivated (DEFRA, 2015).

This high-energy crop is treated as if it were waste; its easy conversion enhances the commercial success of large AD plants. In upscaling AD plants, many have become detached geographically from a specific feedstock source, from low-energy slurry and from the outputs’ end-users. Although the government accepted that such cultivation could be beneficial, it warned: ‘Any intensive production of a single crop could cause environmental concern, whether grown for food, as an AD-specific crop biomass or for transport biofuels’ (DEFRA and DECC, 2011: 13).

Environmental impacts of maize cultivation are controversial. According to critics, ‘[m]aize crops have severe negative impacts on public goods like soils and fresh water’, especially from run-off of pesticides and nutrients. ‘Many farmers are being paid to cause significant harm to these public interests’ (Soil Association, 2015: 2). Thus the financial subsidies for AD were attacked as a perverse incentive for degrading or wasting resources. According to the National Sheep Association, moreover, AD feedstock depletes forage stocks; such ‘problems are caused by scale, either resulting in structures that damage the landscape or a mass change in crop use in particular regions’. These farmers criticized feed-in-tariffs and the Renewables Obligation for incentivizing crop-based bioenergy at large scales (Gastin, 2018).

In promotional visions, AD’s liquid digestate is envisioned as a substitute for chemical fertilizers, which incur significant greenhouse gas emissions through their production and use (WRAP, 2016). Although farm-based AD provides such a substitute, this role is difficult for food-waste outputs converted into digestate, which is expensive to transport, difficult to spread, hard to store, and of varying quality. For these reasons, this potentially valuable resource is treated with suspicion by farmers and has generally remained an economic burden; operators must pay a gate fee for disposing digestate on agricultural land (ADBA, 2016a, 2016b, 2017: 8).

These tensions arose from a specific policy framework. Through a search for techno-fixes, AD became an epistemic-technical challenge of how best to scale up waste conversion while maximizing renewable energy yield (and other resource uses) to achieve low-carbon targets. This imaginary anticipated a future where current waste-generating systems remain largely intact.

## Mechanical and biological treatment: Dual sociotechnical imaginaries

Local Authorities have made efforts to segregate waste for recycling or composting, especially through kerbside collections. Nevertheless, large amounts of recyclables remain in municipal solid waste. Statutory responsibility lies with individual Local Authorities or consortiums of them. To help them fulfil their statutory duty for landfill diversion, the UK’s Private Finance Initiative (PFI) scheme funded a Waste Infrastructure Delivery Programme between 2006 and 2011. This policy created some controversy.

An extra incentive came from the government’s ecomodernist framework. The rising landfill tax was supplemented by the Landfill Allowance Trading Scheme, which imposed penalties for any Local Authority exceeding its maximum allocation of bio-degradable waste in landfills. This allowance could be traded with other Local Authorities, thus creating a market in landfill allowance and further incentivizing landfill diversion.

As a new solution during the PFI scheme, Mechanical and Biological Treatment (MBT) has been sought for several aims: to avoid local incineration (and thus protest), to reduce the output volumes needing disposal or transport, and to avoid the need for kerbside segregation. The PFI scheme funded approximately one-third of the 28 MBT plants.

Minimizing costs and avoiding controversies have been main drivers of Local Authority decisions, even if they are officially justified in environmental terms such as the waste hierarchy (interview, DEFRA, 21.12.2016). In many cases, MBT plants were commissioned as a substitute for source-segregating recyclables or food waste; it would be more expensive to pay for both systems at once. Environmental NGOs criticized some authorities for evading their responsibility to segregate waste, thus yielding poor-quality outputs, which ‘will fetch a lower value in the market’ (FoE, 2008: 5). Indeed, that difficulty later erupted into public protest, as shown below.

**Figures 3.**
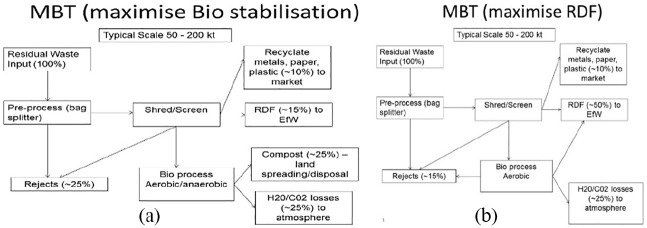
MBT plants with alternative configurations. credit: DEFRA.

MBT plants have basically two design types. One has sought to biostabilize the output for use as a soil improver, facilitating an eco-localization agenda, but this type encountered great operational difficulties. Another type has sought to maximize refuse-derived fuel (RDF), whose lower volume cheapens long-distance transport to seek the lowest gate fee (Figure 3; also Read et al., 2014). This design has been a techno-market fix for systemic waste burdens. Corresponding to dual imaginaries, these designs and their outcomes are analysed in the next two sub-sections, respectively. These correspond to the two lower rows of Table 1.

### Biostabilization design for output reuse

Some early MBT designs were meant to produce outputs for reuse. The process is intended to biostabilize organic waste as a Compost-Like Output (Figure 3a). This would generate much lower methane emissions and could improve soil, even recycle nutrients, according to company publicity. For example, ‘biostabilization aims to reduce the impact on the environment of the putrescible fraction of unsorted waste when landfilled’ (Entsorga, 2016). Another company was contracted for an MBT plant ‘designed to divert over 75% of incoming material into a resource’, some eventually being used for land remediation (Biffa, n.d.). Rather than go to landfill, the municipal solid waste would be turned ‘into fuel, energy and a nutrient-rich soil enhancer’ (see concerns in Let’s Recycle, 2015).

Given the original promises, Friends of the Earth supported MBT biostabilization plants, especially for their flexible scaling and potential to localize waste systems, thus respecting the proximity principle. ‘Plants can be built on a small scale, which would not drag waste in from a large surrounding area’ (FoE, 2008: 3). FoE cautiously supported MBT biostabilization plants, with appropriate financial incentives:

Friends of the Earth believes that the government should introduce a lower rate of landfill tax for waste that has been adequately [bio]stabilised through an MBT process. This would have a significant impact upon the financial viability of MBT technologies in the UK. (FoE, 2008: 6)

The UK government eventually promoted MBT biostabilization for landfill as the best environmental option (DEFRA, 2011b: 14). However, operational difficulties were anticipated by industry experts. ‘[T]he key to a successful operation is process flexibility; as during the operational life of a facility, many changes are likely, for example in waste composition, waste collection methods, material presentation, and so forth’ (Griffiths et al., 2009: 566). A more expensive design could flexibly accommodate such variations. But Local Authorities chose a cheaper design. Consequently, ‘[m]any UK plants will prove costly to remain functional to keep pace with future waste situations’, warned an industry consultant (Read, 2012).

After operations began, some plants found success in selling the Compost-Like Output (CLO) to farmers, for example as a soil remediator (Holder, 2014). Yet difficulties arose with output quality, volume reduction and odour control at many other MBT biostabilization plants (Let’s Recycle, 2015). These problems had several causes: ‘poor build quality, under-sizing of the plant and misunderstanding of the technology’, especially the various process controls, for example air flow, moisture levels, feedstock turning and oxygen levels of the air flow (DEFRA expert, 20.06.2017).

Yet blame has been often displaced onto external causes, especially changes in waste composition of AD feedstock. As an early design assumption, half of municipal solid waste was residual organic in the mid-2000s. But in later years people were wasting less food, and food-waste collection systems became more successful, so kerbside bins had much less organic content than anticipated by MBT designs (interview, CIWM expert, 23.01.2017; also interview, Ricardo-AEA, 06.01.2017).

Given the consequent difficulties of the biostabilization process, the output did not qualify as compost under government criteria. So the available options were little better than landfill disposal. At best, ‘The CLO could be utilised in applications such as landfill restoration or some bulk fill uses’ if complying with the appropriate engineering and quality standards (DEFRA, 2013: 44). Likewise, according to industry experts, CLO would have low quality, suitable for ‘landfill restoration, landscaping or fuel crop production’ – at best, ‘saving the need for higher quality material in these instances’ (CIWEM, 2015: 5).

Conversion difficulties are illustrated by the high-profile failure of Lancashire County Council’s two MBT plants. After a 2002 public consultation drew responses overwhelmingly against incineration, the Council adopted a policy not to incinerate municipal solid waste in Lancashire and then turned to MBT as an alternative. Eventually it agreed to a contract with an Australian company, Global Renewables. Based on its technology’s in-built process control, the company claims to divert 66% of the feedstock from landfill and to produce a high-quality OGM^®^ (Organic Growth Medium), a specific form of CLO (GR, n.d.). Its Lancashire plants were expected to achieve at least 57% landfill diversion – yet diverted <30% in practice (Let’s Recycle, 2013). Serious operational difficulties generated large waste stockpiles and odour problems, leading to complaints by staff and residents (*Lancashire Evening Post*, 2016).

Plants run by other Local Authorities encountered similar difficulties, which led to persistent odours and flies, provoking residents’ complaints, for example at Shanks’ Barrow plant (*The Mail*, 2015). Thus MBT plants elude technological-political control (cf. Moore, 2012: 789), provoking conflicts analogous to incinerators. Yet some protests against new incinerators propose an MBT plant instead (Warne, 2013), thus echoing NGOs’ favourable view from years earlier.

As an extra problem of Lancashire’s plants, the CLO did not fulfil the quality standard as a soil improver for restoring brownfield sites until 2015, and even then at only one-third the quantity expected. The Council blamed the decline in feedstock’s food-waste composition. In 2014, the Council cancelled the contract in order to take control of the operation (*Lancashire Evening Post*, 2016).

A further difficulty came from a change in government policy: the Landfill Allowance Trading Scheme was abolished in 2013. As the Council Leader explained, the government moved the goalposts, so ‘we must take advantage of cheaper options to process Lancashire’s waste’, that is through landfill (quoted in Sanderson, 2016). By 2016, the Council decided to abandon the composting process altogether, on grounds that it was disproportionately expensive for the modest reduction in feedstock volume to CLO and thus the modest cost-savings. Instead, the output would be incorporated into RDF or sent to landfill (Sanderson, 2016).

The Lancashire case exemplifies a widespread failure of technological design across all the UK’s MBT plants (interview, DEFRA, 21.12.2016). As an industry-wide problem, those difficulties arose from over-optimistic claims for technological flexibility.

Promoters of MBT cited the technology’s flexibility, but waste-management contractors have not fulfilled or passed on this benefit. They generally are very restrictive about the feedstock composition requirements, even claiming that compositional variance has caused the treatment process to fail. In reality, contractors do not fully understand the technology that they have chosen and have oversold its benefits. (Personal communication, DEFRA expert, 20.06.2017)

In principle, PFI contracts were meant to remunerate the contractor’s expertise and responsibility to anticipate, avoid or manage any operational problems. When operational problems arise, however, ‘Each party tries to blame the other; a PFI contract is not an appropriate vehicle for risk-sharing’ (interview, Ricardo-AEA, 06.01.2017). This systemic difficulty has arisen from the drive and expectations for a techno-market fix, with the result of blurring or shifting responsibility.

As another difficulty, MBT plants were expected to recover plastics for recycling. In practice, they have carried out a ‘dirty’ plastics recovery, where an energy-intensive process is necessary to clean the plastics. Even so, plastic recyclates have lacked a competitive advantage over virgin plastics, whose price has declined along with the oil price. So outputs have been generally incorporated into refuse-derived fuel (RDF) of a higher calorific value for cement kilns, low on the waste hierarchy. Market incentives have favoured an epistemic know-how valuing energy output per se.

### Bio-drying design for RDF outputs

By contrast with the Local Authorities opting for MBT biostabilization, others opted for MBT-to-RDF plants (Figure 3b), mainly for financial reasons. During the 2006–2011 PFI scheme, EfW plants were widely foreseen as charging RDF gate fees lower than the total of Landfill Allowance Trading Scheme plus the landfill tax, set to rise every year for following next decade. As the main advantage, the MBT bio-drying process lowers the volume output, thus cheapening long-distance transport of RDF to EfW plants.

As it turned out years later, continental incinerators had lower gate fees than in the domestic market, so the UK’s RDF has been mainly exported there, thus further upscaling the material flows and diffusing responsibility for outcomes. Indeed, plants have been designed more recently for exporting RDF to continental plants (interview, CIWM expert, 23.01.2017). The waste burden is effectively shifted across space and time, analogous to some other techno-fixes.

For the MBT-to-RDF type technology, the Shanks company’s Sistema Ecodeco (now E2E) process was adopted for plants of several Waste Authorities (East London, Cumbria, Dumfries and Galloway, etc.). The in-built control system guarantees the output quality, as reported by a UK delegation to Ecodeco plants abroad (MRMC, 2004: 2). RDF combines various heterogeneous materials with a consistent calorific value for combustion in either incinerators or cement kilns (DEFRA, 2013: 28). RDF with high calorific value was foreseen as generating income from cement kilns, by retaining dense plastics rather than extracting them as recyclates. At best, however, RDF outputs have found a lower gate fee or cost-free disposal, not an income (interview, DEFRA, 21.12.2016).

Environmental benefits have been a contentious issue, strongly contingent on the epistemic choice of baseline counter-factual scenario. When some MBT plants were being configured for RDF output, this decision was justified on environmental grounds, namely, that the renewable energy output would replace fossil fuels (CIWEM, 2015: 1–2). But which ones? Incineration seemed environmentally better if the RDF-EfW output replaces coal, the worst baseline, as was the former practice in cement kilns.

According to critics back then, however, such a baseline would be made obsolete by future developments. Coal would be replaced by other fossil fuels, with lower greenhouse gas emissions. Also the feedstock’s calorific value would decline through plastics segregation at kerbside or at MBT plants (Eunomia, 2006). Consequently, plausible future trends would weaken the earlier environmental assumptions favourable to incinerating RDF for greenhouse gas reductions (FoE, 2008: 2).

Eventually the government accepted those criticisms of the RDF design. According to DEFRA, MBT-to-landfill ‘provides the best emissions performance in terms of the treatment/disposal of residual waste’. Yet this best option was contradicted by prevalent incentives: ‘The emissions from waste combustion of non-biogenic material (via any technology including mass-burn incineration) are … not comprehensively reflected in the price of disposal’, thereby creating a financial incentive for RDF production (DEFRA, 2011b: 14, 25; cf. Eunomia, 2008). Thus it blamed anonymous market forces for environmentally worse outcomes.

Given those doubts about the MBT-to-RDF option, the government sought to improve its environmental benefits through heat use. There ‘are potential balance points beyond which energy from waste could perform worse than landfill in carbon terms’, especially if the incineration plant generates only electricity and so wastes the heat. To ensure that incinerators improve carbon balances, it advocated a redesign for combined heat and power (CHP), especially by ensuring that ‘CHP-ready plants’ become ‘CHP in use’ (DEFRA, 2014).

New RDF plants have been built with optimistic assumptions about using the surplus heat for local use and about subsidies stimulating such use. The Renewable Heat Initiative was meant as a market incentive but has had a subsidy rate much lower than for renewable electricity. Heat use has proven elusive, except in some new developments incorporating distribution systems. Retrofits of buildings are difficult and expensive: ‘The disruption involved in connecting a densely populated urban district up to a central source of heat risks adding to the opposition new incinerators routinely attract’ (Ballinger, 2014). Thus surplus heat has found little use, despite an eco-localization imaginary in the UK policy framework.

## Conclusion

Focusing on UK low-carbon waste-energy agendas, we asked: What have been the different visions of societal futures? And how did each vision link technological change with institutional arrangements (continuity or change)? How did the policy framework relate to the different visions? We approached these questions through sociotechnical and economic imaginaries, paying particular attention to how alternatives seek to contest dominant hierarchies (Jasanoff, 2015; Tidwell and Tidwell, 2018). Analysing actors’ strategies can help explain how those hierarchies persist, how alternative imaginations encounter obstacles and thus how they might be overcome.

In our case studies, divergent sociotechnical imaginaries can be understood as techno-market fixes versus eco-localization. Both imaginaries depend on markets and economic resources of some kind. But a techno-market fix emphasizes several market-type elements: financial incentives as the main policy instruments, market competition as the means to stimulate technological improvements and strengthen global competitiveness. Together these elements constitute the nation as a competitive economic space, as a wider economic imaginary for mobilizing resources (cf. Jessop, 2005; Levy and Spicer, 2013).

Implementing the EU’s low-carbon policy, the UK has promoted new markets for technological innovation that could provide low-carbon renewable energy, or treat waste as a resource, or both at once. Financial instruments include landfill taxes, market quotas and subsidies. This policy was intended to reduce greenhouse gas emissions through the conversion process and/or outputs substituting for fossil fuels. Tensions amongst various policy objectives, especially environmental sustainability and economic growth, were reconciled by anticipating future technological innovations, such as ‘advanced’ biofuels that could convert surplus biomass and waste-conversion technologies that could move waste up the hierarchy.

Each technology has had diverse potential designs and societal visions, as summarized in Table 1. Divergent trajectories correspond to rival sociotechnical imaginaries, that is narratives of the public good (cf. Jasanoff, 2015; Jasanoff and Kim, 2009). In the dominant techno-fix imaginary, current centralized systems should be made more resource-efficient through low-carbon technologies (as promised by industry incumbents). In the eco-localization imaginary, a shift to low-carbon systems should localize resource flows, output uses and institutional responsibility (as promoted by civil society groups). These dual imaginaries have informed divergent priorities within sectors such as renewable energy and waste treatment.

The New Labour government’s ecomodernist policy framework combined the rival imaginaries, thus accommodating divergent visions of stakeholder groups, as a stronger basis for political authority. In parallel, low-carbon techno-market fixes gained epistemic authority through anticipatory reasoning. This authority has featured several kinds of know-how: for scaling up low-carbon technologies that previously occupied a specific niche, for maximizing energy yield from them, for more effective conversion processes, and for output uses that could bring waste up the hierarchy. This know-how rendered the policy framework more plausible. Such know-how has resonances with anticipatory governance of emerging technologies (Guston, 2014). Our case study highlights caveats about state-led governance selectively conferring epistemic authority on some imaginaries and thus societal futures.

At the interface of waste treatment and low-carbon strategies, the UK’s ecomodernist framework mandated institutional changes to stimulate various techno-market fixes. The policy framework outsourced waste management and conversion to private-sector contracts with Local Authorities. The latter then faced several difficulties, including higher charges for landfill disposal and protests against incineration. To avoid both difficulties, Local Authorities looked to novel technologies, especially anaerobic digestion (AD) and mechanical and biological treatment (MBT). Both technologies had an interpretive flexibility, broadening their appeal to actors with divergent imaginaries.

Through those technological promises, the ecomodernist framework gained authority and broad acceptance, while obscuring or softening tensions between future visions. From its eco-localization imaginary, Friends of the Earth advocated specific technological designs that could truly bring environmental improvements. But it said little about the policy framework or material-economic basis necessary to implement them. Thus its eco-localization imaginary remained marginal in most waste-energy trajectories.

The UK’s earlier bioenergy agenda drove techno-design towards bioenergy as an input-substitute for incumbent centralized energy systems while marginalizing alternative trajectories. For AD and MBT, financial incentives likewise incentivized technological designs to maximize energy (electricity or gas) for centralized grid systems within the dominant sociotechnical imaginary. Both technologies have been increasingly designed for energy production as global goods, dependent on longer-distance waste flows, distant from the feedstock source or local responsibility.

Such outcomes are rivalrous, preempting other resource uses that would be environmentally more sustainable. Both technologies have had difficulties converting waste into outputs going higher up the hierarchy, at least in a commercially viable way (e.g. compost improving soil, digestate replacing chemical fertilizers, and ‘dirty’ plastics replacing virgin plastics). There are many reasons for the difficulties, including weak market incentives, unstable conversion processes, and low-quality outputs. These issues have precedents in earlier techno-fix strategies that were also aimed at converting waste into materials that would be less burdensome or even valuable. In the UK cases discussed in this article, the latter outputs remained elusive. With the exception of subsidized electricity, material conversions favoured (or had greatest success for) low-value outputs in resource terms. Some processes generated negative-value outputs, whose burdens were shifted across space and time, that is were relocated and/or delayed (cf. LeCain, 2004).

The UK’s ecomodernist framework promoted environmental benefits that were rarely realized in practice. Although it stimulated some waste-management improvements, they neither brought waste very far up the hierarchy nor localized its management. Investment decisions and outcomes largely favoured incumbent interests, reinforcing dominant energy and waste systems, while continuing large-scale waste production. Prevalent trajectories remained low on the waste hierarchy, closer to disposal. Investment priorities eventually marginalized the eco-localization imaginary.

This political-economic outcome has resulted from an ecomodernist policy framework dependent on financial incentives. Institutional decisions anticipated various future trends – output rewards (feed-in-tariffs, Renewables Obligation Certificates, Renewable Heat Incentive, recyclate prices), waste-disposal costs (Landfill Allowance Trading Scheme, landfill tax, gate fees), technological capacities and political obstacles such as local protest. The policy mantra, ‘letting the market decide’, has relegated responsibility to anonymous markets, displacing public accountability of the state and industry (Page, 2012: 940). Indeed, calling for technoscientific innovation is ‘a common way of avoiding change’ (Edgerton, 2006), or at least a change in the incumbent actors.

Let us return to our introduction: Debates over techno-fixes have left ambiguous the relationship between technological and societal change, which may seem like distinctive solutions. As shown here, however, the same technology could be designed and promoted for diverse societal futures, involving sociotechnical changes of different kinds. In our case studies, the specific design was shaped through multi-stakeholder interactions, policy frameworks and resource mobilization for specific trajectories. More generally, some basic technologies have potential trajectories either conforming to the incumbent system or else transforming it (Smith and Raven, 2012).

Socially equitable transitions, including civil society agendas for eco-localization, depend on the necessary conditions for marginal actors to prevail over incumbent ones. These dynamics imply the need for further research on how alternative imaginaries help advocates to mobilize support for their realization. How do they seek economic, institutional and policy support? In some cases, how do alternatives inadvertently become absorbed into the societal vision and policy instruments of the dominant imaginary? Or else how do they contest and even transform it? To investigate such questions, research could carry out longitudinal studies of ‘technology in use’ (Edgerton, 2006; Hyysalo et al., 2018), while integrating theoretical perspectives on sociotechnical and economic imaginaries (Jasanoff, 2015; Jessop, 2005).
